# Aqueous Extract of *Paris polyphylla* (AEPP) Inhibits Ovarian Cancer via Suppression of Peroxisome Proliferator-Activated Receptor-Gamma Coactivator (PGC)-1alpha

**DOI:** 10.3390/molecules21060727

**Published:** 2016-06-03

**Authors:** Chia-Woei Wang, Cheng-Jeng Tai, Chen-Yen Choong, Yu-Chun Lin, Bao-Hong Lee, Yeu-Ching Shi, Chen-Jei Tai

**Affiliations:** 1 Department of Obstetrics and Gynecology, School of Medicine, College of Medicine, Taipei Medical University, Taipei 11042, Taiwan; cwwang@ms4.hinet.net (C.-W.W.); chenyen_318@hotmail.com (C.-Y.C.); 2 Department of Obstetrics and Gynecology, Center for Reproductive Medicine and Sciences, Taipei Medical University Hospital, Taipei 11042, Taiwan; 3 Graduate Institute of Clinical Medicine, College of Medicine, Taipei Medical University, Taipei 11042, Taiwan; 4 Division of Hematology and Oncology, Department of Internal Medicine, Taipei Medicine University Hospital, Taipei 11031, Taiwan; cjtai@tmu.edu.tw (C.-J.T.); f96b47117@ntu.edu.tw (B.-H.L.); 5 Department of Internal Medicine, School of Medicine, College of Medicine, Taipei Medical University, Taipei 11031, Taiwan; 6 Taiwan Indigena Botanica Co., Ltd., Taipei 11031, Taiwan; doclin10@gmail.com; 7 Department of Chinese Medicine, Taipei University Hospital, Taipei 11042, Taiwan; 8 Traditional Herbal Medicine Research Center, Taipei Medical University Hospital, Taipei 11042, Taiwan

**Keywords:** *Paris polyphylla*, ovarian cancer, epithelial-mesenchymal transition (EMT), high glucose induction, peroxisome proliferator-activated receptor-gamma coactivator (PGC)-1alpha

## Abstract

Chemotherapy, a major approach was used in carcinoma treatment, always involves the development of drug resistance as well as side-effects that affect the quality of patients’ lives. An association between epithelial-mesenchymal transition (EMT) and chemotherapy resistance was established recently. We demonstrate in this paper that the aqueous extract of *Paris polyphylla* (AEPP)—a traditional Chinese medicine—can be used in various cancer types for suppression of carcinogenesis. We evaluated the suppressions of EMT and mitochondrial activity by AEPP treatment in a high-glucose (HG) induced-human ovarian carcinoma cell line (OVCAR-3 cells). The mitochondrial morphology was investigated using MitoTracker Deep Red FM staining. Our results indicated that AEPP reduced the viability of OVCAR-3 cells considerably through induction of apoptosis. However, this inhibitory potential of AEPP was attenuated by HG induction in OVCAR-3 cells. The levels of estrogen-related receptor (ERR)-alpha activator and peroxisome proliferator-activated receptor-gamma coactivator (PGC)-1alpha were elevated by HG induction, but were suppressed by AEPP treatment. Down-regulations of cell survival and EMT were oberved in OVCAR-3 cells through suppression of PGC-1alpha by AEPP treatment. These results were confirmed through PGC-1alpha knockdown and overexpression in OVCAR-3 cells. Thus, AEPP can be beneficial for treating ovarian cancer and has potential for development of an integrative cancer therapy against ovarian cancer proliferation, metastasis, and migration.

## 1. Introduction

*Paris polyphylla* is an established herbal medicine, used in fevers, headaches, burns, wounds, and cancers [1]. *P. polyphylla* has been used in Oriental folk medicine for its anticancer activities both *in vivo* and *in vitro* [2]. Numerous natural steroidal saponins isolated from herbs show potential apoptosis-promoting activity against several cancer cell lines [3,4,5]. In addition, *P. polyphylla* treatment can inhibit epithelial–mesenchymal transition (EMT) and invasion in breast cancer [6] and lung cancer [3,4].

EMT results in invasive cells entering the blood stream [7]. Cancer stem cells undergo EMT and then migrate to distant organs [8,9]. During EMT, epithelial cells have decreased expression of epithelial markers (e.g., epithelial keratins, including E-cadherin, occludins, claudins, and desmoplakin) and acquire mesenchymal traits (vimentin, N-cadherin, fibronectin and alpha-smooth muscle actin up-regulation) [10]. Human cancer cells induced to undergo EMT exhibit stem cell-like properties and increased metastatic potential [10]. However, the effects of *P. polyphylla* on migration and proliferation of ovarian cancer cells remain unclear.

Ovarian cancer is a common gynecologic cancer, with the highest mortality rate worldwide. Upon diagnosis more than 70% of ovarian cancer cases are in the advanced stages; currently nonsurgical therapies such as chemotherapy and radiotherapy are the main approaches for ovarian cancer patients [11]. Ovarian cancer is a heterogeneous disease divided into three subtypes: epithelial carcinomas, stromal carcinomas, and germ cell tumors [12]. Among these subtypes, epithelial ovarian carcinomas comprise approximately 85%–95% of all ovarian cancer cases [13,14]. The invasive activity of epithelial tumor cells is based on single-cell migration, such as mesenchymal-type movement [15]. Several metabolic syndromes, including obesity [16] and diabetes [17,18], are associated with ovarian cancer invasion and development. Peroxisome proliferator-activated receptor-gamma coactivator (PGC)-1alpha, a cotranscription factor, causes the activation of mitochondrial activity-associated transcription factors, such as estrogen-related receptor-alpha (ERRalpha) [19], thereby regulating cancer migration and angiogenesis [20]. In the present study, the inhibitory effects of aqueous extract of *P. polyphylla* (AEPP) in OVCAR-3 through regulation of PGC-1alpha expression and mitochondrial function were elucidated.

## 2. Results

### 2.1. Cell Viability

**Figure 1:**
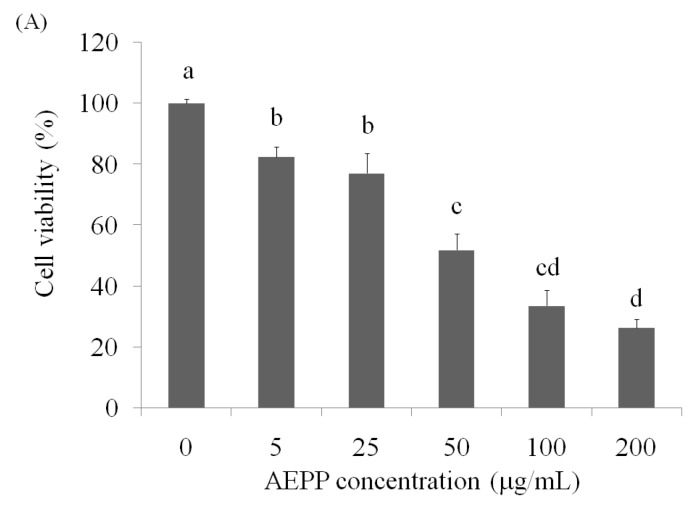

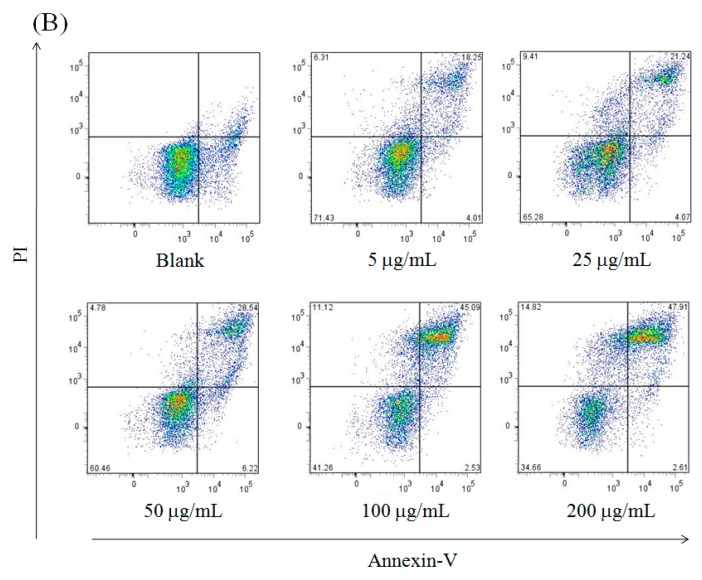
The anti-ovarian carcinoma cells activity of AEPP in OVCAR-3 cells by (**A**) cell viability and (**B**) apoptosis/necrosis measurements. Data were shown as mean ± SD (*n* = 3). A significant difference is shown by different letters between groups (*p* < 0.05).

Growth of OVCAR-3 cells was suppressed by AEPP treatment for 24 h, and the cell viability was <50% at 100 μg/mL (38.72%) compared with the control group (Figure 1A). In addition, apoptosis and necrosis induction caused by AEPP treatment in the OVCAR-3 cells was confirmed by the annexin-V-FITC/propidium iodide (PI) double staining method, which in combination with flow cytometry enabled quantitative assessment of viable (annexin-V-FITC-negative and PI-negative), early apoptotic (annexin-V-FITC-positive and PI-negative), late apoptotic or necrotic (annexin-V-FITC-positive and PI-positive), and dead (annexin-V-FITC-negative and PI-positive) cells. The results clearly indicated that AEPP induced apoptosis in OVCAR-3 cells (Figure 1B; Table 1).

### 2.2. Mitochondrial Fusion/Fission

**Figure 2:**
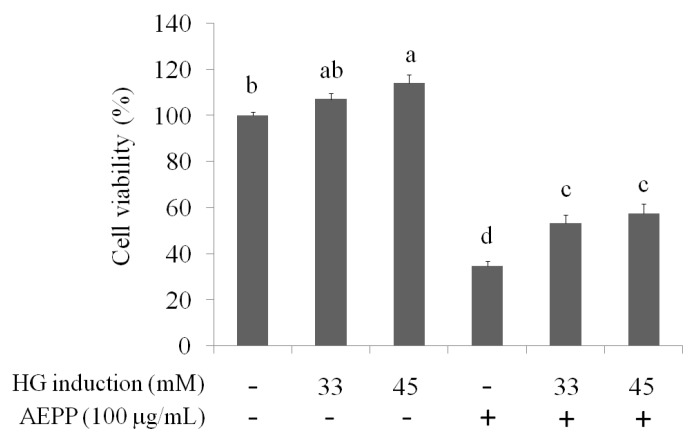
The suppression of AEPP on OVCAR-3 ovarian carcinoma cells induced by high glucose (HG). Data were shown as mean ± SD (*n* = 3). Different letters indicate a significant difference between groups (*p* < 0.05).

**Figure 3:**
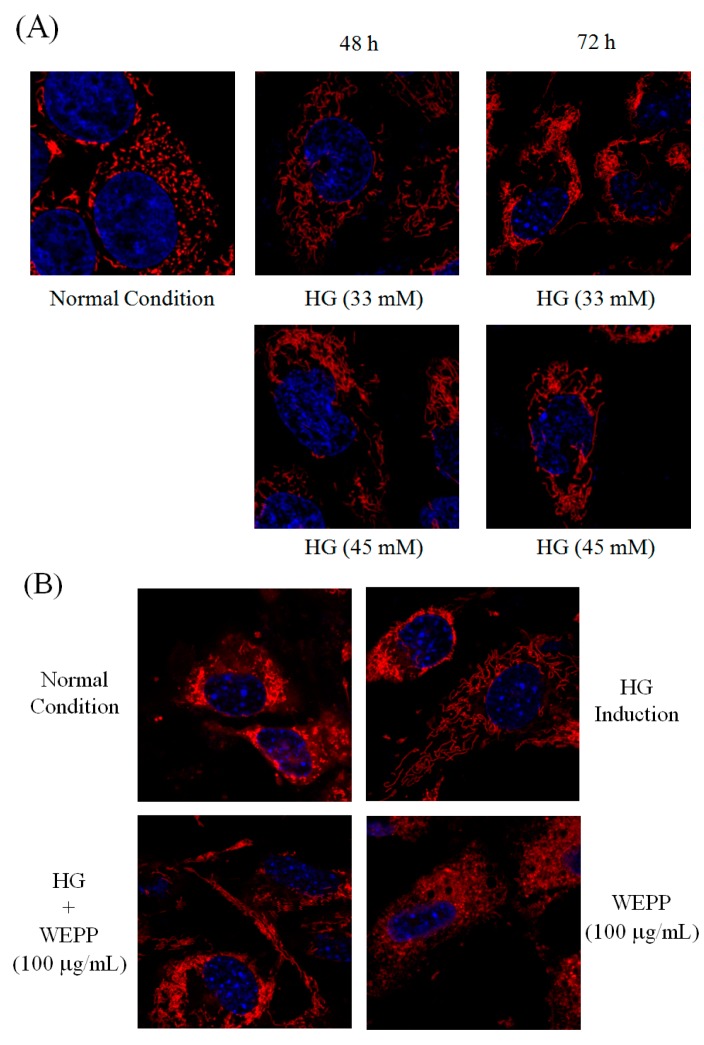
Observation of mitochondrial fission and fusion in OVCAR-3 carcinoma cells treated by (**A**) high glucose (HG) induction for 48 h and 72 h; or (**B**) HG induction with AEPP for 24 h. Fission: mitochondrial fragment; Fusion: mitochondrial network.

Metabolic syndromes are associated with ovarian cancer invasion and development. Here, the effects of AEPP on activation of mitochondrial functon and EMT caused by high-glucose (HG) induction were evaluated. As shown in Figure 2, HG (33 mM and 45 mM) treatment increased cell viability in OVCAR-3 cells compared to the control group. In contrast, the inhibitory effects of AEPP on the cell viability were attenuated in HG-treated OVCAR-3 cells. These results revealed that HG induction can regulate the energy balance and mitochondrial function, resulting in resistance of OVCAR-3 cells against AEPP-induced cell death. Subsequently, the mitochondrial morphology was observed by MitoTracker Deep Red FM staining. Fission and fusion are essential for cell growth, mitochondrial redistribution, and healthy mitochondrial network maintenance. In addition, mitochondrial fission and fusion have prominent roles in disease-related processes such as apoptosis and mitophagy. Mitochondrial fusion (or a mitochondrial network) was markedly observed in both 33 mM and 45 mM HG-induced OVCAR-3 cells after 48 h and 72 h treatment (Figure 3A). Mitochondrial activity in OVCAR-3 cells was attenuated through AEPP (100 μg/mL) treatment resulting in mitochondrial fission (Figure 3B). Taken together, AEPP can inhibit OVCAR-3 cells probably through suppression of mitochondrial activity, but this potential was attenuated by HG treatment.

### 2.3. EMT Marker

**Figure 4:**
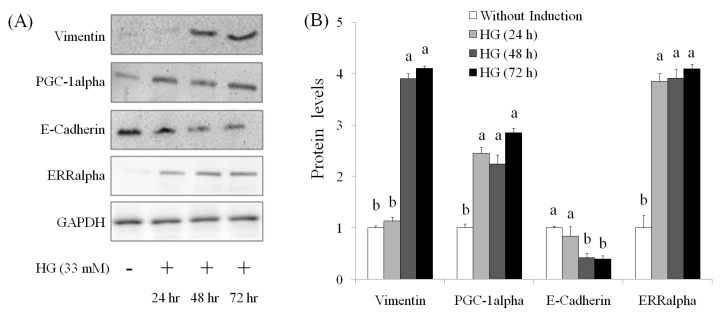
The effects of high glucose induction on (**A**) EMT markers by Western blot and (**B**) the results of quantity analysis in OVCAR-3 carcinoma cells. Data were shown as mean ± SD (*n* = 3). A significant difference between groups (*p* < 0.05) is shown by different letters.

**Figure 5:**
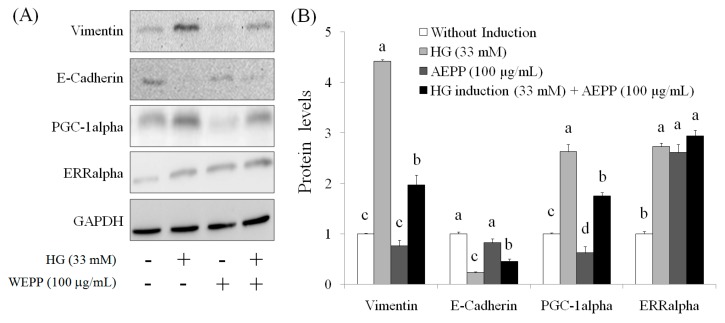
The inhibition of high-glucose-induced (**A**) EMT markers by Western blot and (**B**) the results of quantity analysis in OVCAR-3 carcinoma cells treated by AEPP for 24 h. Data were shown as mean ± SD (*n* = 3). A significant difference between groups in the same protein is shown by different letters (*p* < 0.05).

EMT is a main mechanism of cancer metastasis [21,22]. By undergoing EMT, cancer cells maximize their growth, migration, invasion, metastasis, and drug resistance [23,24]. Therefore, EMT reversal is a potential therapeutic method for suppression of metastasis and chemotherapy resistance [24]. Mitochondrial biogenesis requires coordinated nuclear and mitochondrial DNA expression; the signaling pathways that coordinate the transcription and replication signaling pathways between genomic and mitochondrial DNA require further elucidation. PGC-1alpha plays a key role in mitochondrial biogenesis. PGC-1alpha cooperates with ERRalpha to promote the expressions of multiple nuclear-encoded genes associated with mitochondrial fusion [25]. The levels of vimentin, PGC-1alpha, and ERRalpha were elevated in OVCAR-3 cells treated by HG for 24 h–72 h, but the level of E-cadherin was descreased in HG-induced OVCAR-3 cells (Figure 4). These results suggested that HG induction significantly resulted in EMT in OVCAR-3 cells. By contrast, we observed that AEPP (100 μg/mL) markedly attenuated HG-induced elevations of vimentin and PGC-1alpha levels in OVCAR-3 cells; however, these effects of AEPP were attenuated by HG induction. However, treatment of AEPP did not affect ERRalpha expression in HG-induced OVCAR-3 cells (Figure 5).

### 2.4. Role of PGC-1alpha during Suppression of EMT by AEPP in HG-Induced OVCAR-3 Cells

**Figure 6:**
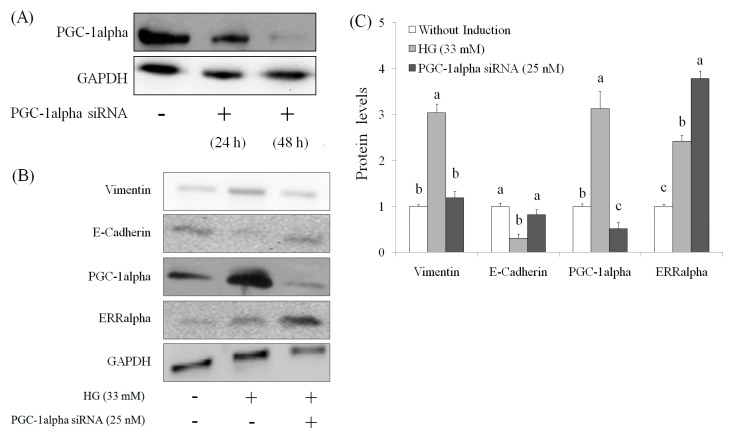
Effects of PGC-1alpha knockdown treatment on epithelial-mesenchymal transition (EMT) markers in high glucose (HG)-induced OVCAR-3 carcinoma cells. (**A**) The cells were treated with PGC-1alpha siRNA for 24 h for suppressing PGC-1alpha levels in OVCAR-3 cells; (**B**) subsequently resulting in HG-induced EMT and (**C**) the results of quantity analysis was carried out. Data are shown as the mean ± SD (*n* = 3). Significant differences between groups for the same protein are shown by using different letters (*p* < 0.05).

**Figure 7:**
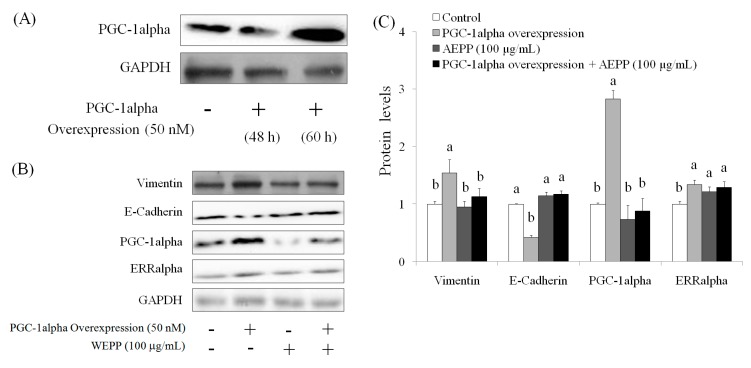
The effects of regulation of PGC-1alpha overexpression (similar to high glucose induction) on EMT markers in OVCAR-3 ovarian carcinoma cells. (**A**) The cells were treated with PGC-1alpha overexpression vector for 60 h for elevation of PGC-1alpha levels in OVCAR-3 ovarian carcinoma cells, and were subsequently treated by AEPP for (**B**) EMT markers, and (**C**) the results of quantity analysis was carried out. Data were shown as mean ± SD (*n* = 3). A significant difference between groups in the same protein level (*p* < 0.05) is shown by different letters.

**Figure 8:**
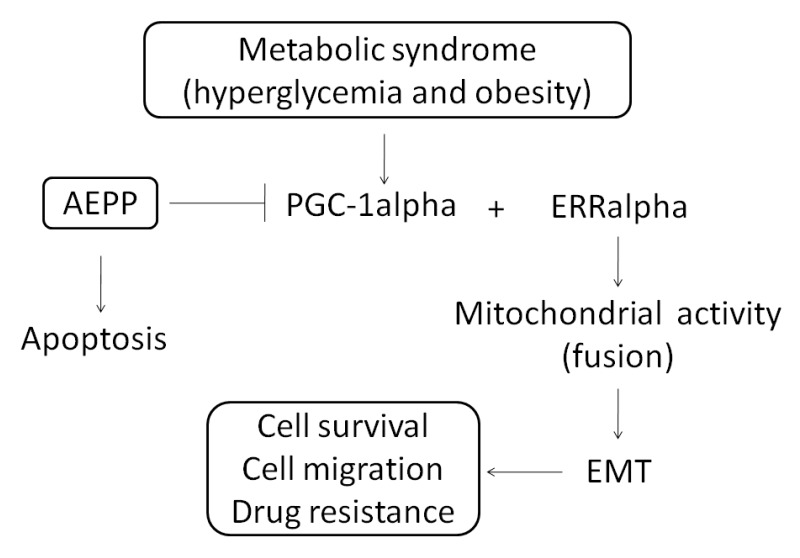
The potential of AEPP against cell proliferation and EMT of OVCAR-3 ovarian carcinoma cells.

The suppression of PGC-1alpha level was found in OVCAR-3 cells (with or without HG induction) by AEPP treatment. Therefore, the role of PGC-1alpha during EMT in HG-induced OVCAR-3 cells treated by AEPP was evaluated. First, small-interfering RNA (siRNA; 48 h) was used for inhibition of PGC-1alpha (Figure 6A). The results indicated that PGC-1alpha knockdown markedly suppressed the elevation of vimentin expression and recovered the expression of *E*-cadherin level in HG-induced OVCAR-3 cells. However PGC-1alpha knockdown did not affect ERRalphain HG-induced OVCAR-3 cells (Figure 6B,C). These results revealed that the loss of PGC-1alpha attenuates EMT—including promotion of vimentin and loss of E-cadherin caused by HG induction in OVCAR-3 cells—and these results were similar to that caused by AEPP treatment. In addition, these effects were confirmed by PGC-1alpha overexpression (similar to HG induction) in OVCAR-3 cells. The results are shown in Figure 7. We observed that PGC-1alpha, vimentin, and ERRalpha levels were significantly elevated after 60-h induction in OVCAR-3 cells; however, this promotion of PGC-1alpha was inhibited through AEPP treatment. Similarly, AEPP treatment inhibited vimentin and elevated E-cadherin levels through suppression of PGC-1alpha in OVCAR-3 cells (Figure 8).

## 3. Discussion

Because of its wide applications in cancer prevention and treatment, public interest in complementary and alternative medicine has been increased worldwide. Traditional Chinese medicine is one of the most common and crucial types of complementary and alternative medicine. Novel molecular prognostic markers, which participate in specific pathways are involved in cervical cancer tumorigenesis and tumor progression. EMT is characterized by an epithelial-to-fibroblast-like morphological and functional shift, which loosens the intercellular junctions and increases cell motility [26]. E-cadherin is a major cell adhesion molecule forming intracellular adhesion junctions in epithelial cells; loss of *E*-cadherin indicates the first stage of cancer cell metastasis [27]. EMT is essential for cancer cell invasion and metastasis [28]; several biomarkers involved in EMT have been identified, including *E*-cadherin, *N*-cadherin, fibronectin, and vimentin [28,29]. Reduced *E*-cadherin levels may enhance EMT and increase cancer cell migration [29,30,31]. In addition, decreased *E*-cadherin expression is associated with poor prognosis in cervical cancer patients [30,31].

In addition to performing metabolic reactions, mitochondria undergo fission and fusion change called mitochondrial dynamics, which has a critical role in regulating cell metabolism, survival, and proliferation [32]. The molecular mechanisms involved in mitochondrial dynamics have been partially elucidated. Fusion unifies the mitochondrial compartments, whereas fission generates morphologically and functionally distinct mitochondria. Mitochondrial fission often occurs early in an apoptotic event [33] and the autophagic process [34]. Mitochondrial fusion is associated with increased cell survival [35]. We observed that AEPP attenuates mitochondrial fusion caused by HG induction, thereby suppressing EMT and apoptosis induction. The role of PGC-1alpha in mitochondrial function was reported recently [36]. Furthermore, ERRalpha regulates mitochondrial function and cancer cell viabilty [37]. An active compound, polyphyllin VII, purified from *P. polyphylla*, can suppress chemoresistance in breast cancer cells [38] and invasion in lung cancer cells [4]. In addition, polyphyllin VII can induce apoptosis and inhibit EMT [22]. Here, we observed that AEPP potentially suppresses the expression of PGC-1alpha (an ERRalpha activator), thereby avoiding ERRalpha activation. In conclusion, HG-induced EMT and cell proliferation in ovarian carcinoma cells was inhibited by AEPP.

## 4. Materials and Methods

### 4.1. Chemicals

AEPP was prepared as follows: *P. polyphylla* was purchased from Taiwan Indigena Botanica Co., Ltd. (Taipei, Taiwan), and 10 g of the herb was extracted with water (100 mL) three times at room temperature for 24 h. After evaporating the solvent through freeze-drying, a residue was obtained and stored at −20 °C. Crystal violet, PI, sodium dodecyl sulfate (SDS), Triton X-100, trypsin, and trypan blue were purchased from Sigma Chemical Co. (St. Louis, MO, USA). Fetal bovine serum (FBS) was purchased from Life Technologies (Auckland, New Zealand). Dimethyl sulfoxide was purchased from Wako Pure Chemical Industries (Saitama, Japan). MitoTracker Deep Red FM was purchased from Invitrogen (Carlsbad, CA, USA). Anti-vimentin, anti-*E*-cadherin, anti-ERRalpha, and anti-PGC-1alpha antibodies were purchased from Santa Cruz (Santa Cruz, CA, USA).

### 4.2. Cell Culture

The human ovarian carcinoma cell line OVCAR-3 was grown in Dulbecco’s modified Eagle’s medium (Gibco BRL, Grand Island, NY, USA) containing 2 mM l-glutamine and 1.5 g/L sodium bicarbonate and supplemented with 10% FBS (Gibco BRL) and 2% penicillin-streptomycin (10,000 U/mL penicillin and 10 mg/mL streptomycin). The cells were cultured in a humidified incubator at 37 °C under 5% CO_2_

### 4.3. Cell Viablitiy

The cytotoxic effects of AEPP against OVCAR-3 cells were measured using a crystal violet staining assay. Cells were seeded on 24-well plates (3 × 10^4^ cells per well) and treated with various AEPP concentrations for 24 h. The medium was then removed, washed with phosphate-buffered saline (PBS), stained with 2 g/L crystal violet in phosphate-buffered formaldehyde for 20 min, and washed with water. The crystal violet bound to the cells was dissolved in 20 g/L SDS solution and its absorbance was measured at 600 nm.

### 4.4. Apoptosis

For detecting apoptosis, floating and adherent cells in the medium were collected after AEPP treatment. Cells were harvested, washed in ice-cold PBS, and resuspended in 200 μL of binding buffer before being incubated in 5 μL of annexin-V-FITC (BD Biosciences, SanDiego, CA, USA) solution and 5 μL of PI at temperature for 15 min in the dark. Subsequently, 300 μL of a binding buffer was added. Cells were analyzed through flow cytometry. Untreated cells were used as double staining controls.

### 4.5. Western Blot

Cells were rinsed with ice-cold PBS and lysed by RIPA lysis buffer with protease and phosphatase inhibitors for 20 min on ice. Then the cells were centrifuged at 12,000× *g* for 10 min at 4 °C. Protein extracts (20 μg) were resolved using SDS-polyacrylamide gel electrophoresis (SDS-PAGE; 200 V, 45 min). The protein bands were electrotransferred to nitrocellulose membranes (80 V, 120 min). Membranes were then treated with a 5% enhanced chemiluminescence (ECL) agent (GE Healthcare Bio-Sciences, New York, NY, USA) in saline buffer (T-TBS) containing 0.1% Tween-20, 10 mM Tris-HCl, 150 mM NaCl, 1 mM CaCl_2_, and 1 mM MgCl_2_ at a pH of 7.4 for 1 h, and then incubated with the primary antibody overnight at 4 °C. Subsequently, membranes were washed three times in T-TBS and bound antibodies were detected using appropriate horseradish peroxidase-conjugated secondary antibodies, followed by analysis in an ECL plus western blotting detection system (GE Healthcare Bio-Science).

### 4.6. Mitochondrial Fission

Cells were treated with 250 nM Mitotracker Deep-Red FM (Invitrogen) for 30 min in a serum-free culture medium. After being washed with PBC twice, nuclei were stained with Hochest 33342 for 10 min. The mitochondrial morphology was observed using a confocal microscope.

### 4.7. PGC-1alpha Knockdown

PGC-1alpha interference of OVCAR-3 ovarian cells was performed with lipofectamine RNAiMAX transfection reagent followed by the protocols provided (Invitrogen). The sequence of specific small interfering RNA (siRNA) for PGC-1alpha was purchased from Santa Cruz. Cell lysates were subjected to western blotting with anti-PGC-1alpha antibody to confirm the inhibition of PGC-1alpha expression.

### 4.8. PGC-1alpha Overexpression

The PGC-1alpha expressing plasmid was purchased from Addgene (Cambridge, MA, USA). Cells were transfected with PGC-1alpha plasmid using lipofectamine-2000 accodring to the manufacturer’s protocol (Invitrogen).

### 4.9. Statistical Analysis

The analysis of variance was used to evaluate the significance of the differences between factors and levels. Comparison of the means was carried out by employing a Student’s *t*-test to identify which groups were significantly different from other groups. The least significant difference was *p* < 0.05.

## 5. Conclusions

*P. polyphylla* is a medicinal herb widely used in Traditional Chinese Medicine. It can considerably suppress human ovarian carcinoma cells by regulating EMT and apoptosis through inhibition of PGC-1alpha, thereby attenuating HG-induced mitochondrial fusion. Our results suggested that AEPP has potential benefits for ovarian cancer therapy.

## Figures and Tables

**Table 1:** Effects of AEPP on apoptosis induction in OVCAR-3 cells.

Group	Surviving Cells	Early Apoptosis	Late Apoptosis	Necrosis
Blank	88.83 ± 2.14 ^a^	7.53 ± 0.84 ^a^	2.41 ± 0.42 ^d^	1.23 ± 0.08 ^c^
5 μg/mL	75.20 ± 1.32 ^ab^	4.14 ± 0.73 ^b^	19.33 ± 1.24 ^c^	1.33 ± 0.11 ^c^
25 μg/mL	62.01 ± 1.75 ^b^	4.89 ± 0.49 ^b^	24.64 ± 1.01 ^bc^	8.46 ± 0.46 ^b^
50 μg/mL	56.80 ± 1.04 ^c^	4.18 ± 0.67 ^b^	29.49 ± 1.48 ^b^	9.53 ± 0.58 ^b^
100 μg/mL	38.72 ± 1.94 ^d^	2.87 ± 0.58 ^c^	48.52 ± 1.22 ^a^	9.89 ± 0.55 ^b^
200 μg/mL	31.23 ± 1.27 ^d^	2.91 ± 0.52 ^c^	50.03 ± 1.45 ^a^	15.83 ± 0.51 ^a^

Data were shown as mean ± SD (*n* = 3). A significant difference is shown by different letters in each column (*p* < 0.05).

## Abbreviations

EMT: epithelial-mesenchymal transition
AEPP: aqueous extract of *Paris polyphylla*
ERRalpha: estrogen-related receptor alpha
PGC-1alpha: peroxisome proliferator-activated receptor-gamma coactivator (PGC)-1alpha
